# Color associations for days and letters across different languages

**DOI:** 10.3389/fpsyg.2014.00369

**Published:** 2014-05-27

**Authors:** Romke Rouw, Laura Case, Radhika Gosavi, Vilayanur Ramachandran

**Affiliations:** ^1^Department Brain and Cognition, University of AmsterdamAmsterdam, Netherlands; ^2^Department of Psychology, Center for Brain and Cognition, University of CaliforniaSan Diego, La Jolla, CA, USA

**Keywords:** cross-modal, synesthesia, color, metaphor, letters, language, days, association

## Abstract

While colors are commonplace in everyday metaphors, relatively little is known about implicit color associations to linguistic or semantic concepts in a general population. In this study, we test color associations for ordered linguistic concepts (letters and days). The culture and language specificity of these effects was examined in a large group (457) of Dutch-speaking participants, 92 English-speaking participants, and 49 Hindi-speaking participants. Non-random distributions of color choices were revealed; consistencies were found across the three language groups in color preferences for both days and letters. Interestingly, while the Hindi-speaking participants were presented with letter stimuli matched on phonology, their pattern of letter-to-color preferences still showed similarities with Dutch- and English-speaking participants. Furthermore, we found that that the color preferences corresponded between participants indicating to have conscious color experiences with letters or days (putative synesthetes) and participants who do not (non-synesthetes). We also explored possible mechanisms underlying the color preferences. There were a few specific associations, including red for “A,” red for “Monday,” and white for “Sunday.” We also explored more general mechanisms, such as overall color preferences as shown by Simner et al. (2005). While certainly not all variation can be explained or predicted, the results show that regularities are present in color-to-letter or color-to-day preferences in both putative synesthetes and non-synesthetes across languages. Both letter-to-color and day-to-color preferences were influenced by multiple factors. The findings support a notion of abstract concepts (such as days and letters) that are not represented in isolation, but are connected to perceptual representational systems. Interestingly, at least some of these connections to color representations are shared across different language/cultural groups.

## Introduction

According to the British Psychologist Chris Arnell, the third Monday in January is the day of the year at which people feel the most depressed. He coined this notion with the term “Blue Monday” (Stone et al., 1985; see also Chow et al., 2005). Interestingly, in Dutch there is an expression of “blauwe Maandag” (blue Monday), indicating a short and meaningless period of time. Color perception is an elementary property of our visual system, based on the receptors' sensitivity to different wavelengths of light. In daily life, however, these simple physiological processes have a wide array of effects, ranging from metaphorical use of color terms in poetry or songs, using certain colors to signal certain connotation (Meier et al., 2004; Meier and Robinson, 2005; Moller et al., 2009; Fetterman et al., 2012), or choosing a particular color for the interior of a house based on the atmosphere that color provides the house. It seems that our ability to “simply” discriminate different wavelengths of light is in our cognitive and affective system interconnected with many different concepts, feelings, associations, and memories (Palmer and Schloss, 2010; Taylor et al., 2013). However, little is currently known about which regularities do or do not exist, and what are the underlying mechanisms explaining such associations. One condition that reflects and augments our understanding of our ability for these types of cross-domain associations is synesthesia.

Synesthesia is a fascinating condition wherein one particular sensation evokes another, seemingly unrelated, sensation. Common types of synesthesia include colors evoked by letters or numbers, or by other ordinal sequences such as the days of the week (Baron-Cohen et al., 1987; Mattingley et al., 2001; Ramachandran and Hubbard, 2001; Beeli et al., 2005; Simner, 2007). Synesthesia can occur in a wide variety of sensory modalities (Novich et al., 2011), exemplified by synesthesias like taste-word synesthesia (Ward and Simner, 2003; Simner and Haywood, 2009; Jones et al., 2011; Richer et al., 2011) or movement-sound synesthesia (Saenz and Koch, 2008). These experiences of synesthetic sensations have a truly perceptual nature and can activate the corresponding sensory cortex (Aleman et al., 2001; Smilek et al., 2001; Nunn et al., 2002; Palmeri et al., 2002; Barnett et al., 2008b). However, other types of synesthesia are more conceptual in nature. For example, for grapheme-color synesthetes, it is not uncommon to associate a particular personality with each letter (Simner and Holenstein, 2007; Smilek et al., 2007a; Amin et al., 2011). Synesthesia is well established as a genuine and “real” condition (Baron-Cohen et al., 1987; Cytowic, 1995; Paulesu et al., 1995; Ramachandran and Hubbard, 2001; Asher et al., 2006; Eagleman et al., 2007; Barnett et al., 2008a).

One of the currently most debated issues is the degree to which this condition is “special” or unique (Cohen Kadosh and Terhune, 2012; Eagleman, 2012; Simner, 2012a,b). In recent years, a body of synesthesia research has gleaned a set of properties that set synesthetes apart from non-synesthetes. In short, synesthetes differ from non-synesthetes in their functional and structural brain properties (for a review see Rouw et al., 2011), as well as in their cognitive profile (for a review see Rothen et al., 2012). Furthermore, synesthetic experiences themselves are traditionally distinguished from normal associations by their specificity, consistency, and automaticity (in the sense that evoking concurrents does not take effort), and by the conscious and perceptual nature of the synesthetic experience (Baron-Cohen et al., 1987; Simner et al., 2005; Simner, 2012a). In contrast, commonalities between synesthetes and non-synesthetes have been found in shared trends in inducer-concurrent associations (e.g., Ward et al., 2006). For example, lighter stimuli fit better with higher pitches, and darker stimuli fit better with lower pitches (e.g., Ortmann, 1933; Karwoski et al., 1942; Marks, 1975, 1978; Hubbard, 1996).

This raises the important question why non-synesthete participants show synesthesia-like mappings across sensations. Note that the question whether the *nature* of an associative experience is shared, is different from the question whether the *specific association* is shared (Rouw et al., 2011). In particular, critical to having synesthesia (Simner, 2012a,b; Deroy and Spence, 2013) is the conscious (explicit), specific and oftentimes perceptual nature of the synesthetic concurrent. Despite absence of these explicit experiences, inter- or intramodal associations across sensations have been obtained in non-synesthetes (Marks, 1987, 1989; Vroomen and deGelder, 2000; Spence, 2011). Thus, perhaps the seemingly subjective and irregular color associations of synesthetes, are somehow related to general regularities in the typical population (see Rich et al., 2005; Simner et al., 2005).

Unfortunately, non-synesthetic cross-sensation correspondences are often measured as associations between scales (e.g., “intensity” mechanisms such as increasing loudness of sounds to increasing luminance (Stevens and Marks, 1965; Marks and Stevens, 1966), or “magnitude” processing, such as the non-synesthetic correspondence between numerical quantities and physical size (Moyer and Landauer, 1967; Henik and Tzelgov, 1982; Foltz et al., 1984; Walsh, 2003; Cohen Kadosh et al., 2007). While these types of correspondences are intriguing in their own right, they do not allow for a direct comparison with synesthetic associations, which are marked by the specificity of the associations, e.g., between a particular pitch and a particular color. In fact, the most common synesthetic concurrents are specific colors.

In this study, we will examine linguistic-color associations in a “general” population (of both non-synesthetes and synesthetes), focusing on letters and days as inducers, and colors as concurrents. These associations were chosen as they constitute the most common synesthetic associations. Main hypothesis of the current study is that patterns of color preferences can be obtained in such a general population as well. Furthermore, the study explores potential mechanisms underlying such letter-color and day-color preferences. First, perhaps the non-synesthetic associations depend on a particular orthographic or phonological property of the letter/word. We therefore compare non-synesthetic letter-color and day-color preferences across different language groups. Second, we asked participants about conscious color associations (to be able to exclude putative synesthetes from our analyses with non-synesthetes). A conscious sensory experience is a key characteristic of synesthesia, which might or might not be related to the patterns of color associations. We contrast these participants without conscious color associations, with participants who do experience colors with (certain) days, letters, and/or numbers. We hypothesize that there are similarities in the patterns of color preferences between the two groups. Third, a set of factors are explored that might help explain general color preferences, such as within-language cross-associations and ordinality effects. Below, we summarize what is currently known about factors underlying patterns of letter-to-color and day-to-color preferences. The current knowledge stems mostly from synesthesia research, but a few studies have compared the synesthetic patterns with general (non-synesthetic) cross-modal correspondences.

### Color preferences to linguistic elements

While synesthetic linguistic-color associations may seem “arbitrary,” studies show that they are not actually completely random. Cross-participant patterns of color preferences are consistently found in synesthetes (Marks, 1975; Rich et al., 2005; Simner et al., 2005; Barnett et al., 2008a). Barnett et al. (2009) found concordance between their study on synesthetic color preferences and those of Rich et al. (2005), and Simner et al. (2005). Studies with synesthetes have found that phonological and orthographical properties, as well as the meaning/conceptual properties of the inducers affect the concurrents (colors) in synesthesia (Barnett et al., 2009; Asano and Yokosawa, 2011, 2012; Brang et al., 2011). Simner et al. (2005) furthermore showed that these biases in color associations are shared between synesthetes and non-synesthetes. For example, the letter A tends to be red and F tends to be green. Letter-color preferences were also shared across language classes: non-synesthetic German participants had significant letter-color correspondences, which showed similarities with the English pattern of color preferences.

A more complex picture appears when the mechanisms underlying the linguistic-color preferences are examined. The regularities can both be based in a “first-order” relationship (a category of color relates to a category of letter/day inducer) and “second-order” relationships (relative differences in inducer relates to relative differences in the concurrent)^1^. As we will explain below, most researchers have studied second-order relationships, although a few findings on first-order of regularities are also reported. We examine both types of relationships, which we view as complementary rather than in conflict in providing explanations for obtained color preferences.

For the sake of clarity, we divide the current literature in two types of data sets on linguistic-color associations in non-synesthetes. The first set relates to number-color associations. As discussed above, quantitative differences between numbers have been related to quantitative changes in color properties. Cohen Kadosh and Henik (2006a) found in non-synesthetes an interference of irrelevant color luminance variations on numerical comparison and vice versa. This effect was replicated in Cohen Kadosh et al. (2008). The same authors used the same paradigm to study synesthete MM in 2006 (Cohen Kadosh and Henik, 2006b). The outcome of this testing partly contradicted their earlier findings; while the synesthete showed the congruity effect between luminance and numerical size, the controls now did not show this effect. Cohen Kadosh et al. (2007) studied nineteen digit-color synesthetes and found that magnitude of the inducing digit was related to luminance (but not to the hue or saturation) of the synesthetic color experience. The non-synesthetic magnitude-luminance effect might be age-dependent; Smith and Sera (1992) showed that two-year-old children, but not adults and older children, associate brightness with small objects and darkness with large objects. In summary, there is a tendency to relate brighter color with smaller numerical value and vice versa. This effect has so far been more clearly and consistently found in synesthetes than in non-synesthetes.

The second set relates to letter-to-color associations in non-synesthetes, which was examined in an elegant study by Simner et al. (2005). In this study, both synesthetes and non-synesthetes showed significant linguistic-color preferences. There was little to no relationship between color preference and alphabetical/presentation order of the letters. Instead, characteristics of the graphemes themselves influenced color preferences; the stimulus letter tends to elicit a color name beginning with the same letter (e.g., b -> blue). This effect was replicated in synesthetes by Rich et al. (2005). These “first-order” relations suggest shared characteristics between synesthetes and non-synesthetes in generating letter-color associations. There are also, however, differences between synesthetic and non-synesthetic preference patterns. First, synesthetes produced a greater depth of color descriptions, longer descriptions, and more color terms (Simner et al., 2005). Second, Simner et al. related properties of the color names to the preferences to those colors. These were three different types of properties (see Supplementary Material); the typicality or ease with which color names are generated according to the Battig and Montague orderings (Battig and Montague, 1969), the frequency (in English) of these color names, and how early these color names are learned in life according to Berlin and Kay's typology (Berlin and Kay, 1969, but see Pitchford and Mullen, 2005). Synesthetes tend to pair high frequency graphemes with both high frequency color terms and with the earliest (i.e., earlier learned in life) color distinctions. In contrast, non-synesthetes showed no effect of grapheme frequency, and preference patterns were not influenced by color name frequency, and the Berlin and Kay (when color names are learned). Non-synesthetes did, however, show an effect of order of material and typicality/ease of generation. Letters presented early in testing are paired with more “typical” (easy to generate, as defined by Battig and Montague) colors. The “ease of generation” ranking did not correlate with color choices of synesthetes.

The role of letter frequency in synesthetic color preferences is somewhat debated. Beeli et al. (2007) found, in German-speaking synesthetes, a positive correlation between letter frequency and saturation (highest linguistic frequency is least saturated). Digit frequency was found to be associated with luminance (with lower-frequency digits generating darker colors). Smilek et al. (2007b) found a relationship between grapheme frequency and luminance, and consistent with Beeli et al., this was stronger for digits than for letters. In a reply to the study by Beeli et al., Simner and Ward (2008) compared this data with their previous findings, by converting the synaesthetic physical color choices from Beeli et al. into the 11 color terms from Berlin and Kay; i.e., black, white, red, yellow, green, blue, brown, orange, purple, pink, and gray. Simner and Ward found that higher-frequency graphemes tended to be paired with higher-frequency color terms, and proposed that certain aspects of the HSL color space (upon which Beeli et al. based their conclusions) may be predicted from color naming. These effects of letter frequency on color preferences were however much weaker (Smilek et al., 2007b) or not present at all (Simner et al., 2005) in non-synesthetes. Watson et al. (2012) found that different letter properties had independent mappings restricted to different dimensions of synesthetic color. Shape was related to hue and letter frequency to luminance. Similarly, Brang et al. (2011) found that more similarly shaped graphemes were related to more similar synesthetic colors. This effect was strongest in individuals who experience their synesthetic color in the outside world (projector synesthetes, Dixon et al., 2004).

Another factor affecting the linguistic-color preference is the phonetic characteristic of the linguistic unit. Rich et al. (2005) found phonetic associations influencing color choice (e.g., the letter i evokes the color white, and the letter j, /dz/, evokes the color orange). A role for phonetic properties of vowels was also found in a meta-analyses performed by Marks (1975). In all these studies, the vowel “a” predominately aroused the colors red and blue, e and i tended to be yellow and white, o tended to be red and black, u was usually blue, brown, or black, and ou (in French) was brown. The author notes that there was no systematic linguistic relation between colors and vowels in their study, (e.g., this would have explained if ou was red (“rouge”) and the e was green (“vert”). Instead, the authors show that vowel-color synesthesia reflects regularities between the sound of the vowel and the hues and brightness of the colors (e.g., brighter colors with higher pitched vowels).

In summary, both synesthetes and non-synesthetes might show non-random patterns of linguistic-color preferences. Interestingly, there is evidence for some highly specific relationships across languages (e.g., the letter A tends to be red and the F tends to be green). In this study, we will probe such specific relationships, and also extend this exploration to days of the week. Furthermore, possible mechanisms underlying color-preference tendencies are explored. These can be language-specific factors; we will test the hypothesis that non-synesthetes use specific relations based on linguistic properties (such as the “r” is red or “b” is blue). Factors may also be cross-language; we will explore if phonological similarities/similar concepts across languages lead to similar color preferences. Some effects are not, or not consistently, obtained in non-synesthetes, such as the frequency effects and the tendency to relate brighter colors with smaller numerical value. Other factors have not yet been studied in non-synesthetes (in particular the effect of ordinality, see below). One factor that did however clearly relate to color preferences in non-synesthetes was “ease of generation” of the color names. This is one of the three factors studied by Simner, and in following of their findings we will test the hypothesis that for non-synesthete this particular factor helps explain preferences of colors assigned to letters or days.

One important factor that has not yet been studied in non-synesthetes, is the sequence effects reported by Rich et al. (2005). In line with the reasoning that color associations reflect the age at which they are acquired, synesthetic colors for days of the week (learned earlier) were less likely to be predicted by the initial letter of the day than were those induced by months of the year (learned later, when the child has already learned to read and write). Similarly, as the conceptual relationship between digits and numbers is learned before the spelling of these number words, the concept of ordering was reflected in the color choice (same choice for “one” and “1”), rather than the spelling of the word (“one” and “O”). Sequence effects are of particular interest because of the strong correlation between cultures that might not share specific letter forms, allowing the relative influence of each effect to be explored in the current participant groups.

### Current study

In a series of experiments, participants are asked to assign particular colors to particular letters, numbers, and days of the week. We expect that non-random patterns of letter-to-color and day-to-color preferences are obtained. Furthermore, we hypothesize that similarities in these color preferences are obtained across languages. Third, we expect that the patterns of color preferences of the participants with no conscious color experience show similarities to the patterns of preferences of the “putative synesthetes,” who indicate conscious color experiences with the days/letters. The study also contains exploratory analyses, examining possible mechanisms underlying obtained color preferences. The setup of the study is as follows. We first present day-to-color preferences. We test each of the three hypotheses: patterns of preferences, similarities in these patterns despite diversity in language, and similarities in these patterns despite diversity in (the presence or absence of) conscious color experiences. Next, mechanisms underlying the day-to-color preferences are explored, in particular the role of overall color preferences (e.g., a tendency to choose “blue,” independent of which stimulus is presented). In the second section, this same sequence of analyses is repeated for letter-to-color preferences: testing the three hypotheses, followed by exploring the role of overall color preferences. The third and final results section explores the role of overall color preferences, across language groups. In particular, what is the role of the three factors examined by Simner et al. (2005): ease of color generation, entry in language (age of acquisition of the color term), and color name frequency.

## Materials and methods

### Participants

#### Dutch

First-year Psychology students at the University of Amsterdam (all Dutch-speaking and living in the Netherlands) received course credit for participating in the current experiments, which were both part of a two-day testing session for all first-year Psychology students. Included were 429 participants, (125 male, 299 female and 4 missing information), mean age = 21 (*SD* = 6). Experiments were approved by the Ethics Committee of the University of Amsterdam, and all participants read and signed an informed consent form before starting the experiment.

#### English

As a match comparison 92 English-speaking participants (living in the USA) were included in the current study (41 male; mean age = 21 ± 2). English-speaking participants were students at the University of California, San Diego (UCSD) and the experiment was approved by the UCSD Human Research Protections Program. Participants completed the survey online on a lab computer after completing an unrelated behavioral experiment in the Department of Psychology and were compensated with credit for experiment participation in psychology courses they were enrolled in.

In contrast to the Dutch-speaking participants (all are Dutch natives), the UCSD participants come from diverse backgrounds. The majority (39) indicated English as their native language, 13 indicated Chinese/Mandarin/Cantonese, 6 Korean, 8 Spanish, 8 Vietnamese, and 18 indicated a variety of other languages. The majority of participants (67) were born in the USA, while 25 participants were not (e.g., 4 in China, 5 in South Korea, and 5 in Vietnam). 21 Participants indicated that both parents were born in the USA, but the majority of participants indicated that either one or both parents were not born in the USA, [e.g., both parents in Vietnam (10), Mexico (6), Iran (3), or China (11)].

#### Hindi

Forty nine Hindi speaking participants were recruited by flyer and word-of-mouth from the general UCSD community and participated in the survey (mean age = 35, *SD* = 7.34). These participants included 23 females and 26 males. Most of the participants (45) were born and raised in India. The others (4) were born in the United States, but were still fluent in reading, writing and understanding Hindi. 22 of the participants currently reside in the United States, while 26 reside in India, and 1 in Malaysia. Participants who reside in the United States moved from India on average about nine years ago and thus have strong cultural roots in India. Participants were given either an online survey or an identical paper version.

#### Non-USA/non-english

The previous three participant groups were tested in order to increase diversity in language/cultural background. As an anonymous reviewer pointed out, none of the three groups are however purely monolingual/monocultural. In particular, all participants are familiar with the English language. This means that obtained effects might be driven by shared English language/ cultural influences that language and culture. Indeed, it is not easy to truly avoid this factor: in these modern times there are fewer and fewer mono-cultural participants (i.e., no influence of other culture/language through movies, television, music or internet). Unfortunately, testing such mono-linguistic/mono-cultural participants would have implied a type of recruiting that was not feasible in the context of the current project. Instead, we examined whether the effects seemed driven by the relative influence of language/culture. This was done by comparing previous results with results pertained in subgroups of participants with a strong cultural influence other than USA/English language.

In this “Non-USA/non-English” participant group, we selected a subgroup of 23 participants within the “English” participant group who were not born in the USA, neither were their parents, and who indicated a language other than English as their native tongue (see also description “English participants” above). Almost all (*N* = 20) of these participants indicated the same native country for themselves and their parents, with a major language of their native country as their native tongue. The native languages in this group were Chinese (*N* = 5), Korean (*N* = 4), Vietnamese (4), Spanish (*N* = 2), and Burmese, Farsi, German, Indonesian, Japanese, Khmer, Swedish, Tagalog. Similarly, in the “Hindi” participant group, we selected a subgroup of 15 participants who were born in India, as were their parents, and who indicated that their mother tongue was not English. All of these native languages are spoken in India, (Hindi (*N* = 5), Bengali (*N* = 2), Tamil (*N* = 2), Gujarati (*N* = 2), and Kannada, Kateli, Punjabi, Urdu). All of these 15 participants were living in India. These two subgroups can be correlated with the Dutch participant group (as these participants are all native Dutch, with Dutch as native tongue). We examine if correlations across languages are still present with these subgroups. Furthermore, the two Hindi/English subgroups are taken together (38 participants total), which is a sufficient number of participants to examine if similar color-to-day and letter-to-day regularities persist in this subset of participants, as compared with the previous findings in Hindi/English participant groups.

### Procedures

This study examines regularities obtained in color preferences, in a “normal” participant group. It originally started with the question whether in a large group of first-year Dutch Psychology students, non-random patterns of color preferences would appear. Interestingly, the students often indicated that they felt that their assigning of a color with a day, letter or number seemed “random.” Results showed however that these ‘random’ answers did indeed reveal regularities. As the non-random patterns were obtained, we then examined if these patterns were similar across participant groups with a diverse cultural and linguistic background. We also asked participants if they had conscious color associations with the stimuli (days and letters). Unfortunately, logistical constraints prevented more elaborate testing of the color associations of these participants. Still, the question whether participants experience colors is by at least some researchers taken as the defining feature that separates synesthetes from non-synesthetes (Deroy and Spence, 2013), and other researchers have challenged the standard test of using consistency to determine synesthesia (Simner, 2012a). No doubt, this is an interesting and important factor by itself in understanding patterns of color-preferences. We therefore focus on the role of this factor on the color preferences. As more elaborate testing would likely exclude at least some of these participants as “synesthetes,” we separate those participants with conscious color experiences from those who do not, by referring to these groups as “putative synesthetes” vs. non-synesthetes.

#### Dutch

First, participants read a short description of synesthesia, followed by probe questions designed to reveal any possible synesthesia; participants were asked whether to them, days of the week, certain letters, and/or certain numbers have a certain color. If participants responded “yes” to any of the probe questions, they were asked to describe the color they associated with each item (e.g., day of the week), as precisely as possible. Those who responded “no” (non-synesthetes) were instructed to still assign a color: although to you days/letters/numbers do not have a color, we would still like to know which color you chose with a certain day/number/letter. Each participant was tested twice (test-retest). In the retest, the participants indicating no colors with days, letters or numbers received additional instruction to remember which color they had provided last time, and to provide as accurately as possible the same color description as before. Participants indicating they did experience color were simply asked to provide their color association again (no instruction to remember or match their previous description). These same questions were repeated for several categories: 7 days of the week, all 26 letters of the Latin alphabet, and numbers (1–14 as well as the numbers 20, 50, 100, 250, 4000, and 20.000). In the retest, presented at least two weeks later, the same categories were repeated but with the items rearranged in a random order. The whole test was presented in Dutch.

#### English

As in the Dutch survey, participants read a short description of synesthesia on the computer-screen, followed by questions about synesthesia and then questions about their color associations. The main stimuli where all days of the week, and letters printed in lower case or upper case [A, S, U, K, T, W, n, b, s, l, F, H, I (capital i), D, i, E]. While the UCSD students saw 16 stimuli in total, two letters were presented twice, in upper case and in lower case (s and i). As participants assigned highly similar colors to capital and small font, and because we would not compare them in subsequent analyses with the other language groups, the two lower case versions of the letters i and s were not included in further analyses, leaving 14 letters in the analyses. For the follow-up analyses, participants saw 10 words and 10 non-words (scrambled versions of each of the real words), and 11 numbers. Putative synesthetes were asked to provide the color of each item (e.g., day of the week), as precisely as possible and then to rate how strongly the color was perceived on a scale of 1–100. Non-synesthetes were asked to provide a color that they associated with the item. The colors of letters and days were analyzed and can be compared with those from the Dutch sample. (The color associations with words raised new questions that are now explored in a separate study).

#### Hindi

All participants were presented with the same questionnaire as the English-speaking participants. 30 Surveys were administered on paper and 19 online. Participants were asked about their color-associations for days of the week (Monday to Sunday), numbers (1, 2, 3, 4, 5, 6, 7, 8, 9), letters (phonetic translations of the letters in the English test) and words (again, the word-colors are not further analyzed). For each of the 14 letters in the English test, a phonetic translation was provided (see Table 1). To more clearly define the exact sound on which the letter should be translated, we provided an English word defining pronunciation of each letter. These were: wear, some, zoo, cat, tin, wobble, know, best, lamp, fun, hush, these, dumb, and ache. This translation was then send to an independent Hindi-speaking evaluator for back translation. If this resulted in a different letter than the original English letter, the letter was again translated. This process was repeated until no discrepancies between translation and back translation remained. The whole survey was presented in Hindi.

**Table 1:**
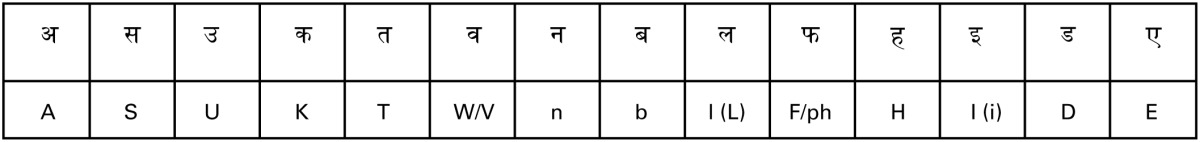
**Hindi letters used in these experiments, and their matching phonetic sounds**.

In Hindi, each of the seven days of the week is apportioned to one or more Hindu gods or goddesses, and several days have folklore or ritual fasting associated with them. The seven days are named after the “celestial bodies” of the solar system: Raviãra: Sunday, day of Sun; Somavãra: Monday (day of Moon), Mañgalvã: Tuesday (day of Mars), Budhavãra: Wednesday (day of Mercury), Guruvãra: Thursday (day of Jupiter), Sukravãra: Friday (day of Venus), and Sanivãra: Saturday (day of Saturn).

#### Coding

Each color-item association made by each participant was coded by two research assistants according to the coding schema of Simner et al. (2005) and Rich et al. (2005). These coding schemes includes eleven basic colors: red, yellow, green, blue, purple, pink, orange, brown, black, gray, white (Berlin and Kay, 1969). In accordance with these earlier studies descriptions of other colors, were recoded according to a fixed pattern. For example, “apricot” was coded as light orange. Responses that could not be classified as a color (e.g., clear or transparent), or responses that included several colors (e.g., green and blue, or rainbow) were excluded. Turquoise was coded into green.

## Results

### Prevalence of explicit color associations

#### Dutch

***Questionnaire***. In the Dutch questionnaire a test—retest was administered. We identified participants who said “yes” to the questions on explicit (conscious) color associations both times. Of the 457 participants, 47 indicated in both the test and retest that they perceived colors with days (10%), 7 indicated colors with letters (2%), and 12 indicated colors with numbers (3%).

#### English

***Questionnaire***. Of the 92 participants, 15 (16%) indicated that they saw colors with days of the week, 10 indicated colors with letters (11%), 9 indicated colors with numbers (10%).

#### Hindi

***Questionnaire***. Of the 49 participants, 15 (31%) indicated that they saw colors with days of the week, 3 indicated colors with letters (6%), 2 indicated colors with numbers (4%).

### Color preferences in dutch, english and hindi subgroups: days

In this section we present the patterns of color preferences for days of the week. In each of the three language groups, non-random color preferences were present for each of the days of the week. In the next sections, we first correlate results across languages, and then examine if these results still hold in a subgroup of participants selected on their non-English, non-USA background. Next, we examine results separately for participants indicating color-day associations (“putative synesthetes”) and those not indicating color-day associations (non-synesthetes). The last subsection of this paragraph examines the effect of overall color selection biases on color-item preference patterns (across the three language groups).

#### Cross-language

First, we examined cross-language consistencies in these day-color preferences. The number of participants choosing a particular day-color combination (*N* stands for number of color-to-day categories used by participants in that test) were correlated between the three participant groups. The distribution of these variables was not normally distributed (Kolmogorov-Smirnov of Dutch and Hindi color choices *p* < 0.1 and of English color choices *p* < 0.05), thus non-parametric correlations are calculated in this results section (“Days”). The Spearman's correlations showed consistencies in the order of day-color preferences across these languages: Hindi—English [*rs*_(64)_ = 0.51, *p* < 0.001]; Hindi—Dutch [*rs*_(66)_ = 0.54, *p* < 0.001], and English—Dutch [*rs*_(73)_ = 0.84, *p* < 0.001]. These correlations suggest that participants did not randomly chose colors for weekdays. While some days do not show a clear first-choice color, there are a few days with high similarity of most important color choices across languages. Table 2 shows similarities in the primary and secondary color preferences across languages. The strongest cross-language effects are obtained with Monday and Sunday: in each language group the strongest preference for Monday is red or blue, and for Sunday it is white or yellow. See Supplementary material for an overview of color choices per color category for these days, for each of the three language groups.

**Table 2:**
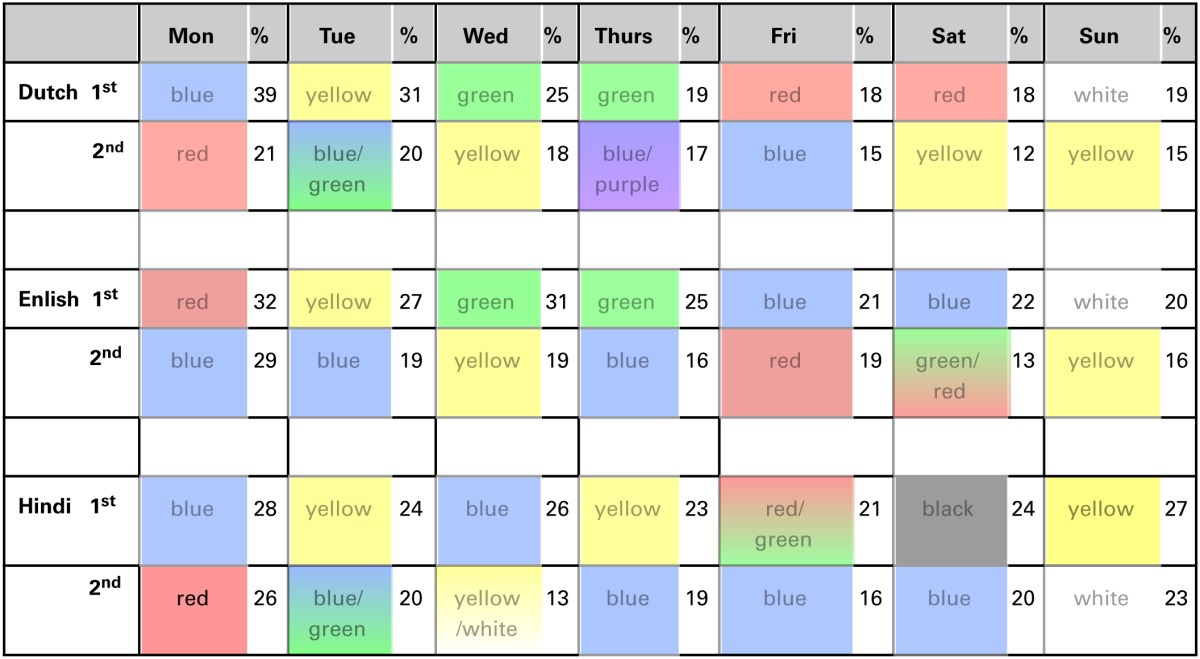
**Consistency between languages for day-color preferences**.

*The highest and second-highest color preferences are presented for each day of the week for the Dutch, English, and Hindi speaking participants. Next to each day-color, the percentage of participants choosing that particular combination (of the total participants assigning valid colors choices for that day). If two colors have an equal number of choices, both are presented in one square*.

### Non-english native language subgroups

We selected a subgroup within the English-speaking participants who were not born in the USA, whose parents also were not born in the USA, and who indicated that their mother tongue was not English (see “Participants”). Similarly, in the Hindi group we selected a subgroup whose native language was not English, were currently living in India, were born in India, as were there parents. These two groups allowed to examine if the obtained effects were due to an underlying shared language/cultural influence (in these participant groups, USA/English). We examined the degree to which effects are diminished in these subgroup with a relatively weak influence of USA culture/English language. The color-word preferences of this group of participants were correlated with the color-word preferences in the Dutch subgroup, who also do not have a USA/English background. Both subgroups still showed significant correlations with the Dutch group; English [*rs*_(77)_ = 0.46, *p* < 0.001] and Hindi subgroup [*rs*_(77)_ = 0.36, *p* < 0.001]. The correlation between the two subgroups is however only marginally significant [*rs*_(77)_ = 0.19, *p* < 0.1]. Next, we examined the degree to which in these participant groups the main effects (as reported in the previous section) were still present. We collapsed the two subgroups (Hindi and English participants with relatively little English/USA background), as else there would be too few counts or participants in these item-by-item comparisons. This subgroup has 38 participants (note that sometimes invalid answers were given, so not all color choices to a particular day add up to 38). For the color-to-days, the main effects were a red and a blue color preference with Monday, and white and a yellow color preference with Sunday. The first and second choice for Monday are still blue (*N* = 8, 26%) and red (*N* = 7, 23%). Similarly, the first choice for Sunday is still white (*N* = 6, 20%). The second choice is now red (*N* = 5, 17%) as much as yellow (*N* = 5, 17%). Thus, the main effects in day-to-color preferences are largely preserved in these participants with diverse cultural and linguistic backgrounds. Overall, even though none of the participants included in this analysis had English as their native language or USA as their cultural background, they still shared regularities in their color-to-day preferences. However, the effects were stronger in the combined/more inclusive groups, and a possible influence of USA culture or English language (e.g., through movies, music and internet) cannot be excluded. The results are still clearly present when nobody in the participant group has an English/USA background, raising the question of why across participants with different language/cultural backgrounds, certain days tends to evoke a particular preferred color association.

### Explicit vs. implicit color preferences

Next, cross-language correlations were examined separately for participants indicating color-day associations (“putative synesthetes”) and those not indicating color-day associations (non-synesthetes). This analysis revealed similar patterns of day-to-color preferences between languages, with the consistencies somewhat stronger for non-synesthetes (participants indicating that to them days of the week do not have color). The correlation between Hindi and Dutch speaking participants was significant for non-synesthetes [*rs*_(67)_ = 0.54, *p* < 0.001], but not for putative synesthete participants [*rs*_(67)_ = 0.31, *p* < 0.011]. The correlation between Hindi and English speaking participants was a bit higher in non-synesthetes [*rs*_(63)_ = 0.52, *p* < 0.001], than in putative synesthetes [*rs*_(41)_ = 0.46, *p* < 0.003]. This same pattern was obtained for non-synesthete English and Dutch speaking participants [*rs*_(73)_ = 0.84, *p* < 0.001] vs. putative synesthete participants [*rs*_(49)_ = 0.63, *p* < 0.001]. We offer two cautionary notes regarding these analyses. First, no thorough screening of putative synesthetes took place, so the “synesthete” group could in fact be a mix of synesthetes and non-synesthetes. Second, there are relatively few participants in each synesthetic group.

As can be expected based on these correlations, within each language group the putative synesthetes and non-synesthetes were very similar in their day-to-color preferences. Significant correlations for color-to-day preferences were obtained between putative synesthetes and non-synesthetes within the Hindi [*rs*_(71)_ = 0.58, *p* < 0.001], English [*rs*_(45)_ = 0.63, *p* < 0.001], and Dutch [*rs*_(90)_ = 0.86, *p* < 0.001] speaking participants.

There are similarities across participants in day-to-color preferences. These similarities are found both across languages. Furthermore, they are not dependent on the trait of synesthesia, if anything the cross-language effects were stronger in non-synesthetes than in participants indicating explicit day-to-color associations (putative synesthetes). What factors underlie these strong correlations? To examine this question, we first consider the role of overall color preferences. For example, participants might exhibit a general bias to choose red more often than gray; such overall preferences could skew the day-to-color preference patterns.

### Overall color preferences

This section presents exploratory analyses examining possible underlying mechanisms to the day-to-color preferences. First, to examine the effect of overall color selection biases on color-item preference patterns, we calculated overall color selection preferences in the three participant groups. Next, we related these preferences to the three factors studied by Simner et al. (2005), as explained in the Introduction: 1. order of entry of color into language (e.g., “white” is learned earlier in life than “yellow”); 2. color name frequency (in English language); 3. ease/order of color generation (in spontaneous generation of color words, some are produced earlier and more often than others). The sequence in which colors are ordered according to color entry and color name frequency did not correlate with overall color preferences in English, Hindi or Dutch speaking participants. In contrast, the color frequency did correlate with ease of color generation in all three participant groups [*rs*_(11)_ ranged between 0.84 and 0.96, *p* < 0.001]. Thus, the most frequently produced colors were those ranked highest for typicality/ease-of-generation (Battig and Montague, 1969).

This raises a question of whether the cross-language correlations are driven by this overall bias. The analyses performed on day-to-color preferences per participant group were repeated while controlling for the factor “ease of color generation.” This analyses showed that the correlations were still significant: English and Hindi [*rs*_(61)_ = 0.32, *p* < 0.05], English and Dutch [*rs*_(61)_ = 0.76, *p* < 0.001], Dutch and Hindi [*rs*_(61)_ = 0.40, *p* = 0.001].

The overall number of choices for a color was related to ease to generate these colors. What, then, is the influence of these general color preference on the patterns of day-to-color associations we obtained? Are the correlations amplified by general color biases within the participant group? We next examined day-to-color preferences while controlling for overall color biases. First, the overall color selection frequencies (across days) were calculated for each color in each language group. Then, for each language group, and for each day, a chi-test was computed to determine whether the number of selections for a given color was significantly different from the expected value (based on the weekly percent selection of that color in that language group). Following the previous results, we examined whether the red and a blue color preference for Monday still holds, and the white and yellow color preference for Sunday. Table 3 shows only the chi values that are at or below 0.05 (and for which the color is chosen more often than on average across all weekdays). Note that this is an exploratory study only, thus the variety of results in the whole table is mostly meant to create hypotheses for follow-up studies.

**Table 3:**
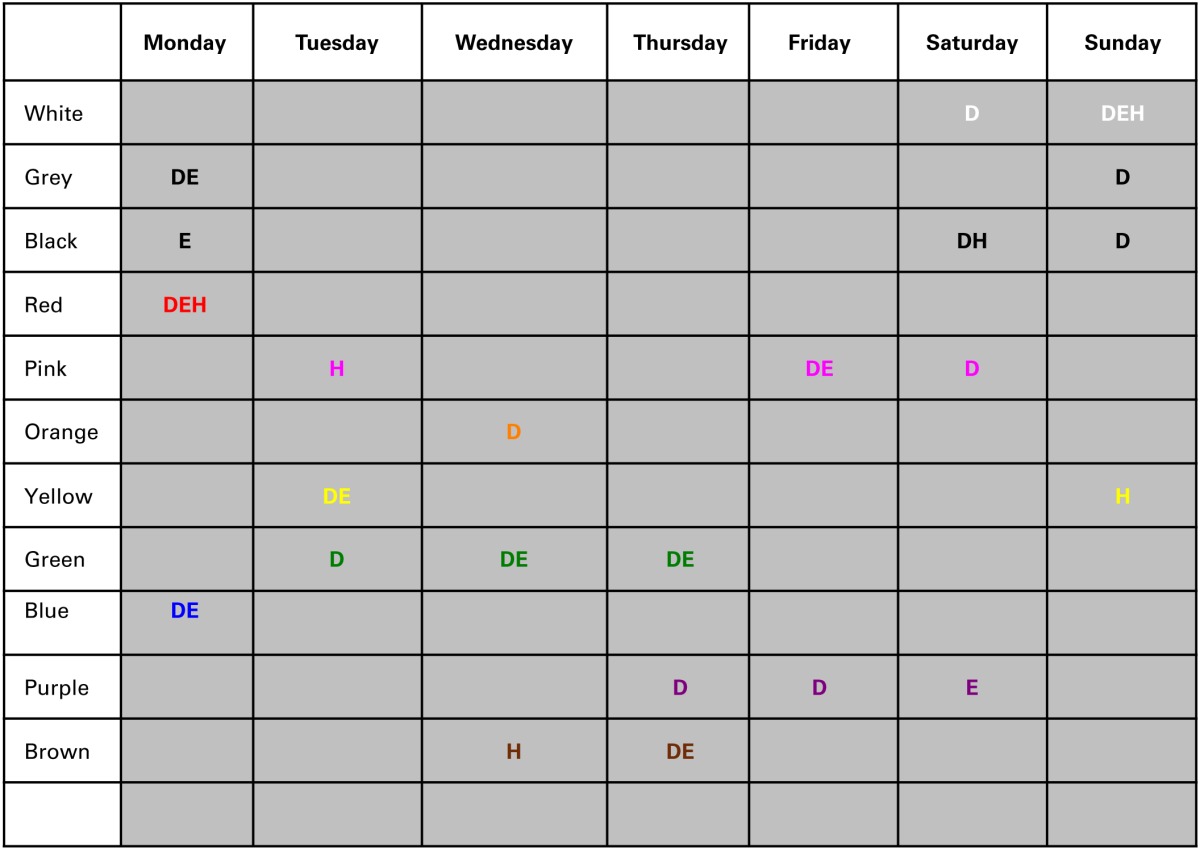
**Colors for each of the weekdays that were significantly different from expected values (in a chi-test), in the Dutch (D) English (E) and Hindi (H) speaking participant groups**.

*Expected valued is based on the percentage selection of each color in each language group*.

Some clear effects are obtained in this analysis. First, red is assigned to Monday in all language groups at a significantly higher rate than to the other days of the week. Interestingly, red is also chosen at a significantly higher rate only for Monday in all language groups. The other effect shared by all language groups is assigning the color white to the Sunday. The association between yellow and Sunday was not confirmed by this analyses and thus may in fact be reflecting an overall color bias. Finally, blue is now only found related to Monday. This effect is present in Dutch and English but not in Hindi speaking participants. Possibly it reflects the notion of “Blue Monday,” which exists in Dutch (“blauwe maandag”) as a saying, and as an urban myth (the most depressing day of the year) in English. As far as we know such expression does not exist in Hindi. The rest of these (exploratory) tests showed that the only other day related to white is the Saturday (in the Dutch speaking group). Furthermore, primary colors black, white and gray are only given to Monday or week-end days. Different from the previous analyses that did not take overall color bias into account, we now also find that days in the middle of the week (Tuesday, Wednesday, Thursday and also Friday) are matched to secondary and/or more complex colors, namely purple, pink, orange, yellow, green, and brown.

### Summary: day-to-color preferences

In this section we present the patterns of color preferences for days of the week. In each of the three language groups, non-random color preferences were present. We obtained cross-language correlations on the patterns of color preferences. Next, we explored possible underlying mechanisms. We first established that these results still hold in a subgroup of participants selected on their non-English, non-USA background. The cross-language correlations were still present, and specific main effects were also still present: a preference for red or blue color with “Monday” and white color preference to “Sunday.” Next, we examined results separately for participants indicating color-day associations (“putative synesthetes”) and those not indicating color-day associations (non-synesthetes). Synesthesia does not appear to be the underlying reason for the cross-language similarities; instead the cross-language effects were stronger in non-synesthetes than in putative synesthetes. The last subsection of this paragraph presents exploratory analyses on the effect of overall color selection biases on color-item preference patterns (across the three language groups). It showed that while “ease of color generation” affects color choices, taking out this factor still results in significant cross-language correlations. Furthermore, taking out overall color preferences still shows particular day-to-color main effects (e.g., red Monday and white Sunday).

### Color preferences in dutch, english and hindi subgroups: letters

In this second subsection, we show patterns of color preferences for letters. Analyses are with the 14 letter stimuli presented to all language groups. These letters were chosen from the latin alphabet to include both vowels and consonants, letters from both the beginning and end of alphabet, and letters with both curved and straight-shapes. For the hindi questionnaire, letters were translated based on their phonological properties (see methods section). We first examine letter-to-color preferences across the three language groups. We then examine whether cross-language similarities in letter-color preferences still hold in a subgroup of participants selected on their non-English, non-USA background. Next, results are examined separately for participants indicating color-day associations (“putative synesthetes”) and those not indicating color-day associations (non-synesthetes). The last subsection of this paragraph examines the effect of overall color selection biases on color-item preference patterns (across the three language groups).

#### Dutch, english and hindi subgroups

First, a cross-group analysis was performed on color preferences with letters. English and Hindi letters were matched on phonology (but of course differed in orthography), see Table 1. The distribution of these variables (reflecting the number of particular colors assigned to particular letters, in each of the three language groups) was not normally distributed (Kolmogorov-Smirnov test of Dutch, Hindi and English color choices were all significant, *p* < 0.005), thus non-parametric correlations are calculated in this results section (“Letters”). English-to Hindi color associations correlated significantly [*rs*_(136)_ = 0.43, *p* < 0.001]. English and Dutch letters had exactly the same shape, and similar (but not exactly the same) phonology. Again significant correlations [*rs*_(167)_ = 0.68, *p* < 0.001] were obtained. Dutch and Hindi had different orthography and similar (but not exactly matched) phonology, but still color preferences correlated [*rs*_(139)_ = 0.59, *p* < 0.001].

As can be viewed in Table 4, the (first and second) strongest color-to-letter preferences have different cross-language consistency for the different letters. A few specific effects, however, do arise. First, there is a strong tendency to choose red for the letter/sound “A.” This effect is present in all language groups. Second, there is a tendency to choose blue for the letter/sound B. See Supplementary material for the percentage color choices for the letter A and letter B in the three language groups. Perhaps this is because of linguistic priming; both the English and Dutch color words (“Blue” and “Blauw”) start with the letter B. (In Hindi the word blue starts however with “*N*”). Other preference effects are weaker, and not expected: for example, it is not clear to us why the letter T has a green or blue color.

**Table 4:**
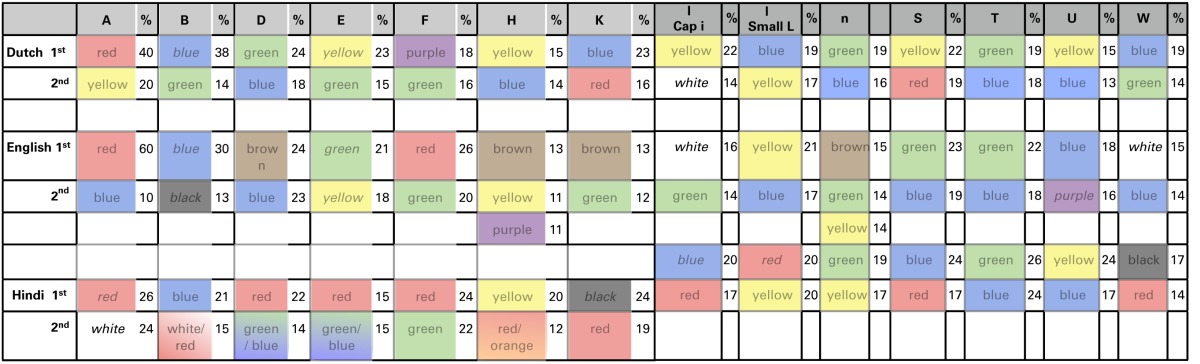
**Consistency between languages for letter-color preferences**.

*The highest and second-highest color preferences are presented for each letter (or phonetic equivalent), for the Dutch, English, and Hindi speaking participants. Next to each letter-color, the percentage of participants choosing that particular combination (of the total participants assigning colors for that day). The color word is presented in italic, if the letter matches with the color word (i.e., it is the first letter or the vowel of the color name)*.

The “Red A” replicates earlier findings (Rich et al., 2005; Simner et al., 2005; Barnett et al., 2009). In this study, the effect is replicated in different participant groups with different linguistic backgrounds; Dutch, English and Hindi. The preference for the color red for the Hindi letter pronounced as “A” indicates that it is not the shape, nor the particular (latin) letter identity. One possible explanation is that the red color is somehow connected with the position of the letter in the alphabet: the first letter in each of the three alphabets gets a “signal” color. While not all calendars are typeset in the same way, for many people (in USA, Holland, India), Monday is the start of a new workweek. Similarly, the red color of the Monday could be to mark it is the first day of the (work)week. To explore this explanation, the preferred color for the number 1 was determined for each of the three language groups. In the Hindi subgroup, of all number-color combinations, the highest preference was obtained for red “1” (14 participants indicated a red “1,” 33% from the total of 42 provided colors to the number 1). In the English group, red and blue were most often chosen (each 17 participants, 18.5% of the choices). The Dutch participant group behaved differently, with white (22%) and yellow (21%) as most common color choices. The preference of white with the number 1 is in line with previous findings with English and German speaking synesthetes (Beeli et al., 2007; Barnett et al., 2008a) and English speaking synesthetes as well as non-synesthetes (Rich et al., 2005). Thus, while there is some support for the idea of the red color of “A” or “Monday” as signaling the first (ordinal) item in a sequence, the evidence for associating the number “1” with red is rather mixed.

#### Non-english native language subgroups

We looked at the subgroup of participants in both the Hindi-speaking and English-speaking groups who were not born in USA and did not have parents born in the USA, and whose native language was not English (for details see the “participants” section). If an overall USA/English language influence is driving the shared color-preferences, the effects should be diminished in the current subgroups. The letter-color preferences of these two subgroups correlated [*rs*_(153)_ = 0.84, *p* < 0.001]. Furthermore, the color preferences of both groups correlated with the Dutch group; English participants [*rs*_(154)_ = 0.46, *p* < 0.001] and Hindi participants [*rs*_(153)_ = 0.56, *p* < 0.001]. The Hindi subgroup also correlated with the color preferences of the Dutch participant group [*rs*_(153)_ = 0.56, *p* < 0.001]. Thus, while none of the participants included in this analysis had English as their native language or USA as their cultural background, they still clearly showed shared regularities in their color-to-letter preferences. Furthermore, these correlations were of comparable size to the ones obtained in the previous (overall) analyses. Next, we examined whether the main color-to-letter regularities reported above still persist in this combined subgroup (English and Hindi who are non-native English/USA). These participants showed a clear and strong letter-to-color preference for the letter A (*N* = 18, 51%). Next, there was a tendency to choose black with the letter K (*N* = 10, 28%), for blue with the letter “b” (*N* = 9, 28%), and green for T (*N* = 9, 24%). This is in line with the findings in the native Dutch group (see Table 4), where the strongest effects were a red A and a blue B.

#### Explicit vs. implicit color preferences

Cross-language consistencies were examined in putative synesthetes (participants indicating explicit color associations with the letters/days) vs. non-synesthete participants. While orthography was different, the Hindi- Dutch correlation in non-synesthetes were significant [*rs*_(139)_ = 0.26, *p* = 0.002]. It was however not significant between putative synesthetes [*rs*_(139)_ = 0.09, *p* = 0.27]. Similarly, the Hindi - English [*rs*_(134)_ = 0.44, *p* < 0.001] correlations were significant in non-synesthetes, but again not significant for putative synesthetes [*rs*_(71)_ = −0.05, *p* = 0.70]. The English and Dutch alphabet has the same orthography (but sometimes different pronunciation) and showed significant correlation in non-synesthetes [*rs*_(79)_ = 0.31, *p* = 0.006] and in putative synesthetes [*rs*_(79)_ = 0.29, *p* = 0.009].

While between languages the correlations were higher for non-synesthetes than for putative synesthetes, there is an overall effect of between-participant consistency in letter-to-color choices. In each individual language group the putative synesthetes and non-synesthetes were very similar in their day-to-color preferences: English [*rs*_(84)_ = 0.42, *p* < 0.001]; Dutch [*rs*_(182)_ = 0.56, *p* < 0.001] and Hindi [*rs*_(140)_ = 0.23, *p* = 0.007].

#### Overall color preferences

Next, the overall color preferences (collapsed across letters) were calculated per language group. Color preferences of the English speaking group correlated highly with the Hindi group [*rs*_(13)_ = 0.91, *p* < 0.001] and with the Dutch group [*rs*_(14)_ = 0.95, *p* < 0.001, *N* = 14]. The Dutch overall preferences correlated highly with the Hindi preferences [*rs*_(13)_ = 0.91 *p* < 0.001].

To examine the nature of the overall color bias, these color preferences were correlated with Color Frequency, Color Ease, and Color Entry. Frequency of Color Name did not correlate with color preference, for the English participants (the factor was extracted originally from English language, see Simner et al., 2005) the correlation was [*rs*_(11)_ = 0.05, *p* = 0.89]. Color entry specifically correlated in Hindi subgroup only, [*rs*_(10)_ = 0.65, *p* = 0.04] (this is significant both for putative synesthetes and non-synesthetes). Ease of Color Generation correlated with preferences in all groups, English [*rs*_(11)_= 0.85, *p* = 0.001], Dutch [*rs*_(11)_ = 0.90, *p* < 0.001], and Hindi [*rs*_(11)_ = 0.89, *p* = 0.003].

This raises a question of whether the cross-language correlations are driven by this overall bias. The analyses performed on color-letter preferences per participant group were repeated for letters, while controlling for Ease of Color Generation. This analyses showed that the correlations were still significant: English and Hindi [*rs*_(129)_ = 0.331, *p* < 0.001], English and Dutch [*rs*_(129)_ = 0.60, *p* < 0.001], Dutch and Hindi [*rs*_(129)_ = 0.28, *p* = 0.001].

#### Chi-test

As previously performed in the analyses of colored days, we examined the pattern of color preferences with letters while taking overall color bias for letters into account. For each language group, and for each letter, a chi-test was computed to determine whether the number of selections for a given color was significantly different from the expected value (based on the percent selection of that color in that language group). We examined whether the main effects in the previous analyses still hold in this control analysis; these were red/A, blue/B, and a green or blue T. This analysis only showed that the “red A” and “blue B” effects are still present in Dutch and English, but not Hindi, subgroups. The blue or green association with T is not present. Table 5 shows only the chi values that are at or below 0.05 (and for which the color is chosen more often than on average across all letters). As with Table 3, please note that these results are exploratory only. These patterns will need to be confirmed by further research on larger samples of individuals in these language groups.

**Table 5:**
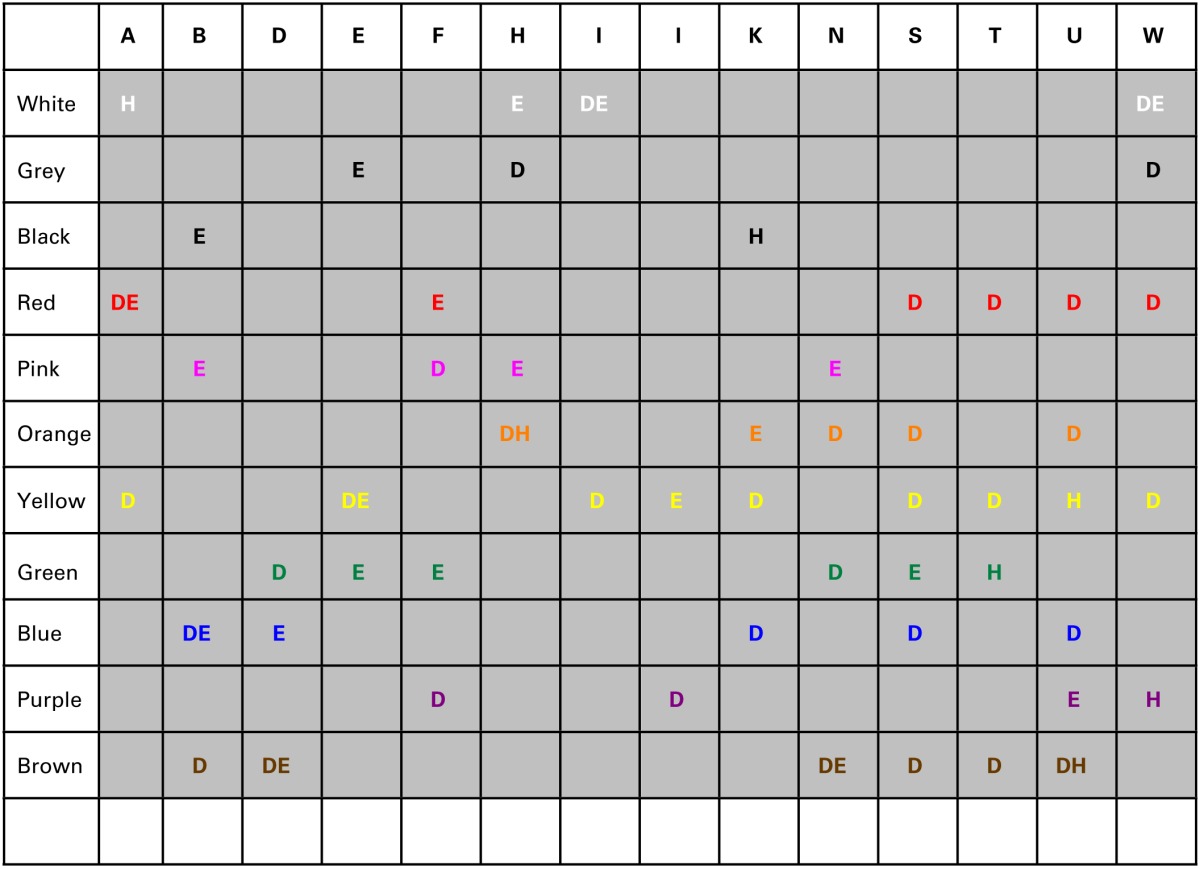
**Colors for each of the letters that were significantly different from expected values (in a chi-test), in the Dutch (D) English (E) and Hindi (H) speaking participant groups**.

*Expected valued is based on the percentage selection of each color in each language group. Only the chi values that are at or below 0.05 are presented*.

### Summary: letter-to-color preferences

In this section we present the patterns of color preferences for letters. We found cross-language similarities in color choices, even with the Hindi language group were letters were matched based on phonology. While most letters do not show a clear and strong “first choice” color across languages, a few specific preferences do appear. Particularly strong is the cross-language preference for red with the letter A, but also a preference of blue with B and of green or blue with T. Next, we explored possible underlying mechanisms for non-random color preferences. We first established that both the cross-language correlations and the obtained specific “first-choice” effects still hold in a subgroup of participants selected on their non-English, non-USA background. Next, we examined results separately for participants indicating letter-to-day associations (“putative synesthetes”) and those not indicating these associations (non-synesthetes). Synesthesia does not appear to be the underlying reason for the cross-language similarities. Again, while there are similarities in color choices between putative synesthetes and non-synesthetes, the cross-language regularities were stronger in non-synesthetes than in putative synesthetes. The last subsection of this paragraph presents exploratory analyses on the effect of overall color selection biases on color-item preference patterns (across the three language groups). It showed that while “ease of color generation” affects color choices, taking out this factor still results in significant cross-language correlations. An exploratory analyses showed that some specific effects, such as green or blue with T, might be explained by overall color preferences. There are however still specific “red A” and “blue B” effects.

### Overall color preferences (across language subgroups)

We next explored the role of overall color preferences in the assignment of colors to days and letters. The data are collapsed across the three language groups. In this section we examine the role of three factors possibly influencing color biases, following the analyses of Simner et al. (2005): ease of color generation, color entry, color name frequency'. As in Simner et al., the color name frequency was measured with English color words (Appendix I). Based on the previous results, we expect that the factor “ease of color generation” is particularly important for non-synesthetes, as compared with the putative synesthetes (who indicate to experience colors with days or letters).

#### Days

We first looked at the color responses provided with days of the week, across language subgroups. The data were collapsed to letter-color preferences over the three language groups. In these collapsed data, we calculated the percentage of number-color choices separately for putative synesthetes and non-synesthetes. These variables indicate, per day, the ratio of how often a certain color is chosen (from all color choices for that day). These two variables, percentage day/color choice for putative synesthetes and non-synesthetes, were not normally distributed [Kolmogorov-Smirnov; *Ks*_(85)_ = 0.16 *p* < 0.001 and *Ks*_(85)_ = 0.14, *p* < 0.001], respectively), thus a non-parametric correlation was calculated. The correlation showed a high correspondence between the synesthetic and non-synesthetic color choices, [*rs*_(85)_ = 0.87, *p* < 0.001].

Next, we examined whether the color choices were related to ease of color generation, and found this significant for both non-synesthete [*rs*_(77)_ = 0.73, *p* < 0.001] and putative synesthete [*rs*_(77)_ = 0.68, *p* < 0.001] participants. Entry in language (age of acquisition of the color term) showed a significant, but weaker, correlation with both and non-synesthete [*rs*_(77)_ = 0.28, *p* = 0.01] and putative synesthete [*rs*_(77)_ = 0.33, *p* = 0.003] participants. There was no significant relationship with color name frequency. As this latter factor might be a more language-specific effect, we separately examined the English participant group, but again found no correlation between color name frequency and color preference in putative synesthetes and non-synesthetes (*p* > 0.1).

A possible alternative explanation for the cross-participant similarities is that ease of color generation underlies the correlations. Therefore, the correlations were repeated with ease of color generation partialled out; still a strong correlation was maintained between “putative synesthete” and non-synesthete color choices [*rs*_(74)_ = 0.80, *p* < 0.001].

Thus, there are regularities in day-to-color preferences, and these are shared between the participants who indicate that to them, days have color, and those who do not experience color with days. For both participant groups, overall color preferences to days are related to ease of color generation and entry into language.

#### Letters

We then explored the overall color biases with letters, across language subgroups. Kolmogorov-Smirnov tests also indicated that neither ‘putative synesthetic’ [*Ks*_(182)_ = 0.20, *p* < 0.001] nor “non-synesthetic” letter-to-color percentages [*Ks*_(182)_ = 0.12, *p* < 0.001] were normally distributed, therefore a non-parametric correlation was used. This showed similar overall color-to-letter preference for putative synesthetes and non-synesthetes [*rs*_(182)_ = 0.69, *p* < 0.001].

Non-parametric correlation analyses showed a strong effect of “ease of color generation” on the color choices of non-synesthetes [*rs*_(154)_ = 0.77, *p* < 0.001] and a significant, but weaker, effect with the putative synesthetes [*rs*_(165)_ = 0.33, *p* < 0.001]. Furthermore, “color entry” correlated with non-synesthete color choices [*rs*_(140)_ = 0.28, *p* = 0.001], but not those of the putative synesthetes. There was no effect of “color name frequency” on the choices of color categories of non-synesthetes or putative synesthetes. As color name frequency was measured with English color words, a separate analyses on the “English” participant group was performed. This however again showed no correlation between color name frequency and color preferences, either in the non-synesthetes [*rs*_(160)_ = 0.05, *p* = 0.51] or in the putative synesthetes [*rs*_(85)_ = 0.15, *p* = 0.16].

If ease of color generation influences both synesthete and non-synesthete color choices, does this explain the obtained correlation between the two participant groups? A partial correlation was run, showing that synesthetic and non-synesthetic color choices still correlated [*rs*_(151)_ = 0.671, *p* < 0.001] when controlling for the factor “ease of color generation.”

#### Summary: overall color preferences

There was a clear effect of “ease of color generation” on the color choices, somewhat stronger with the non-synesthetes but also significant for putative synesthetes. There is a weaker effect of “entry in language” (age of acquisition of the color term) on both participant groups, and no effect of “color name frequency”. Note however that in previous results this effect was related to specific properties of the color that were not measured in the current study, as the naming procedure in assigning colors only defines categories of the color hue. The cross-participant correlations (putative synesthetes and non-synesthetes) show that there are regularities in the data. These regularities are still present if the overall color preference based on “ease of color generation” is partialled out.

## Discussion

In the current study we find statistically significant, non-random patterns of day-to-color preferences and letter-to-color preferences in non-synesthetes. Moreover, there are similarities in these patterns across three language groups: Dutch, English and Hindi language. The third hypothesis was also confirmed: there are similarities in the patterns of color preferences for non-synesthetes as for the putative synesthetes in this study. While clearly not all variation is explained by cross-language associations, and random (or unexplained) influences are also present in the color choices, results suggest regularities as well. As discussed in the Introduction, regularities can both be based in a “first-order” relationship (a category of color relates to a category of the letter/day inducer) and “second-order” relationships (relative differences in inducer relates to relative differences in the concurrent). As expected, our results indicate a role for both types of regularities, in a complementary rather than excluding manner. A few specific day-to-color preferences appear to be particularly strong and consistent, such as red/Monday, blue/Monday, and white/Sunday. The day-to-color preference patterns were furthermore shared over different language groups, and also present in participants with a cultural background other than USA/English. In letter-to-color preferences, we also obtained certain specific preferences. The strongest effect is the red/A bias, which has previously been reported in English (Marks, 1975; Simner et al., 2005) and is now extended to English, Dutch and Hindi language subgroups. Furthermore, biases for blue/B, and green or blue T were obtained. The overall letter-to-color preference patterns are at least to some extent shared across the language groups (and were still present in participants with a cultural background other than USA/English). The similarities in the patterns of color preferences between Hindi and English/Dutch indicates that orthography of the letters, and linguistic properties of the weekday names, are not the only or defining characteristics of the color preferences. As we will explain below, it is likely that different factors are simultaneously at play in generating color preferences. As expected, there are shared biases in color preferences of participants with conscious color experiences with letters (“putative synesthetes”), and participants who do not report such a conscious color association (non-synesthetes). Exploring the patterns of color preferences, we also obtained differences between the groups, as the participants without any explicit color associations showed stronger cross-language similarities (both with letters and days) than the participants who indicated to have conscious color associations with this material. Perhaps not surprising (and in replication of the results of Simner et al., 2005), the factor “ease of color generation” influenced color preference, and this effect was effect was somewhat stronger for the non-synesthetes (compared with putative synesthetes). Importantly, partialling out this factor still led to significant correspondences in cross-language color associations.

Exploratory analyses examined the factors driving the color preferences. Possibly, the preference of “red” with Monday or the letter A is to mark the start of a sequence (of the workweek/ of the alphabet). In line with obtained linguistic effects in synesthetes (Rich et al., 2005, but see Marks, 1975; Simner et al., 2005), there might be effects in non-synesthetes as well. An example of such linguistic-specific effects is the preference of blue with “b” (Simner et al., 2005), which in the current study was obtained both in English as in Dutch (the Dutch word for blue is “blauw”) but not in Hindi (Hindi word for “blue” does not start with a b). These are not the only factors influencing color preference, however. Non-synesthetes showed an overall bias to select colors that are easy to generate (Battig and Montague, 1969). To a lesser extend, there was also a bias to choose colors that were learned earlier in life (Berlin and Kay, 1969).

Mechanisms that allow associations between seemingly unrelated sensations appear to be common to us all. This is illustrated by the mere existence of figurative speech and metaphors, prevalent in art but also ubiquitous in daily life. These cross-modal correspondences resemble synesthesia, in terms of making the same type of associations. One famous example is the association of made-up round vs. spiky shapes with their imaginary names: (“maluma” vs. “takete,” Köhler, 1947; “bouba” vs. “kiki,” Ramachandran and Hubbard, 2001). Another well-known finding is the connection between numbers and space in the spatial-numerical association of response codes (SNARC, Dehaene et al., 1993; Fias et al., 1996). Cross-modal mappings exist across different types of information (e.g., pitch /musical intervals to brightness or shapes (Karwoski et al., 1942; Marks, 1974; Hubbard, 1996) or taste/flavors to sounds (Simner et al., 2010). Furthermore, increased intensity tends to be intuitively related to increased intensity across different types of media (e.g., loudness/brightness/size/ (Marks, 1974, 1987). For a review of cross-modal correspondences see Spence (2011). Clearly, the similarity between synesthetes and non-synesthetes exists in the type of associations made. The current study suggests that while participants without conscious color experiences often commented during the experiment that their answers were “completely random,” in fact they were not. As synesthetes, they show patterns or biases in their patterns of color-to-concept associations. Furthermore, non-synesthetic biases in cross-modal correspondences can even be found cross-language/culture. We suggest that the biases that are likely to influence a particular color chosen with a particular letter or day might be more generally shared across cultures and languages as well as across individuals.

One limitation of the current study is a degree of similarity between cultures; Dutch and American participants share a shared influence of a culture in the structure of the work-week and weekend, and associations such as Sunday as a day of rest or religious day. Hindi-speaking participants, currently living in America, may share some of these influences, depending on the duration of their time in the United States. While the questionnaire itself was completely in Hindi and the participants were native speakers, this might have influenced their color choices. The commonalities in letter-sounds could be due to some shared cultural/language aspect, or translation of English influences into the native (Hindi) language. Alternatively, the commonalities are related to cross-language sound-to-color associations which are either biological or learned pre-literacy. Spector and Maurer (2008, 2011) found that some shape-to-color associations are present pre-literacy and depend on the shape of the letter, while others are later learned literacy effects (such as the red A and green G). These findings show the influence of shape of the letter (Brang et al., 2011), the current results furthermore show an influence of sound of the letter. Given possible shared language/cultural influences, it would be interesting to see if correspondences can also be found in other, additional languages/cultures.

In this and previous studies, similarities are obtained in the cross-linguistic associations in synesthetes and non-synesthetes. What does this tell us more generally about how “different” synesthetes are? As we have argued before (Rouw et al., 2011; “trait vs. type”), similarity between synesthesia and non-synesthetes lies in the exact correspondences made between apparently unrelated modalities (“type”). What is different between these “normal” cross-modal correspondences and synesthetic experiences (“trait”). Most important is the phenomenology of the experience, which is more explicit/conscious, precise, and consistent in synesthetic than in non-synesthetic experiences. Presumably, synesthetic concurrents (but not non-synesthetic color associations), are really qualitative experiences, rather than mere (semantic) associations. In line with these reports, functional and structural brain differences between synesthetes and non-synesthetes are found in sensory brain areas (Nunn et al., 2002; Hubbard et al., 2005a; Rouw and Scholte, 2007; Hupe et al., 2012). Second, non-synesthetes have to exert effort to generate associations (there were many comments during the experiment from the non-synesthetic participants; that it was a silly task, and their color choices felt “completely random”). In contrast, for synesthetes the associations are described as “automatic” in the sense that it takes little effort to produce the concurrent when presented with the inducer. This characteristic of synesthesia might very well be related to the overwhelming evidence (Esterman et al., 2006; Muggleton et al., 2007; Weiss and Fink, 2008; Jäncke and Langer, 2011; Rouw et al., 2011; Specht, 2012) of the role of the parietal cortex in synesthesia. This is most commonly interpreted as reflecting the “hyper binding” present in synesthetes (although the parietal cortex has also other roles (Hubbard et al., 2005b; Cohen Kadosh et al., 2008). Importantly, next to these behavioral and brain differences between synesthetes and non-synesthetes, there is evidence for a genetic predisposition for synesthesia (Asher et al., 2009; Brang and Ramachandran, 2011; Mitchell, 2011; Tomson et al., 2011) and synesthesia has been found “running in the family” (Barnett et al., 2008a). The studies on the patterns of color preferences show how the nature of the color experiences are different: the (putative) synesthetes show more diverse, more specific, more consistent color associations than the non-synesthetes (Palmeri et al., 2002; Hubbard and Ramachandran, 2005; Eagleman et al., 2007). Furthermore, non-synesthetes are more influenced by general factors as evidenced in the current study (see also Rich et al., 2005; Simner et al., 2005; Barnett et al., 2009) by stronger cross-language effects, as well as a stronger effect of the factor “ease to generate color name.” Thus, evidence so far indicates that the differences are found when examining the trait of having synesthesia (what underlies having these experiences). While similarities are found when examining which particular types of associations are made. The trait provides a predisposition to develop unusual experiences (Rouw et al., 2011). The types are shared biases leading to non-random patterns of associations. Furthermore, these biases are shared among different (language/culture) groups. Whether nature or nurture (or a combination of the two) underlie these associations, is a topic of future research.

As a final note, previous studies have examined synesthesia in non-English languages. Simner and colleagues (Simner et al., 2011) found that both native and non-native Chinese speakers with synesthesia experience colors with Chinese characters. Again, these linguistic-to-color associations show (non-random) patterns of associations. For at least some of the synesthetes, the color choices of the characters and words are influenced by the initial letters. Asano and Yokosawa (2012) studied synesthetic coloring of Kanji characters, which are acquired later in life than other types of graphemes in Japanese language. Synesthetic colors were found related to phonology and meaning more than to orthography. At least some influence from earlier learned languages on the later languages were obtained. Indeed, age of acquisition could influence color-letter associations (Asano and Yokosawa, 2011). Barnett et al. (2009) examined synesthetic colors with months, days, and numbers in bilingual synesthetes. The (across-language) words had more similar colors based on commonalities in visual form across languages. In particular months (as compared with numbers or days) had similar colors based on beginning with the same first grapheme, the authors suggest that this is possibly because months are learned later in life when the child is already learning to read and write. Again, these findings with synesthetic colors suggest a mixture of effects influencing linguistic-color associations. This does not necessarily mean that one finding conflicts with another: the different influences can point at different moments in life influencing color preferences, earlier for perceptual involvement of low-level perceptual processing (e.g., Brang et al., 2011) and later for more conceptual, semantic or linguistic influences (e.g., Barnett et al., 2009; Asano and Yokosawa, 2011, 2012).

While analyses of putative synesthetes vs. non-synesthetes should be interpreted with care, the results show similar patterns of color preferences between these groups, both cross- and within-language. Color choices are most likely driven by multiple factors, including linguistic/phonetic influences, orthographic properties, and sequence/ordinality effects. More general effects are also present, such as ease with which the color name is generated. We propose that both synesthetic and non-synesthetic associations show that abstract concepts are not represented in isolation, but are connected to other representational systems. The presence of connections across-representation is independent of whether participants report conscious associations (as occurs in synesthesia) or not. Interestingly, we found that patterns of day-color and letter-color associations are present in non-synesthetes, and biases in color preferences are shared across different language/cultural groups.

### Conflict of interest statement

The authors declare that the research was conducted in the absence of any commercial or financial relationships that could be construed as a potential conflict of interest.

## Footnotes

^1^We thank an anonymous reviewer for suggesting this distinction
